# Supplementary figures and images for: Diversity and distribution of sediment bacteria across an ecological and trophic gradient
Source: PLoS One. 2022 Mar 21;17(3):e0258079. doi: 10.1371/journal.pone.0258079 (PMC8936460; doi:10.1371/journal.pone.0258079)

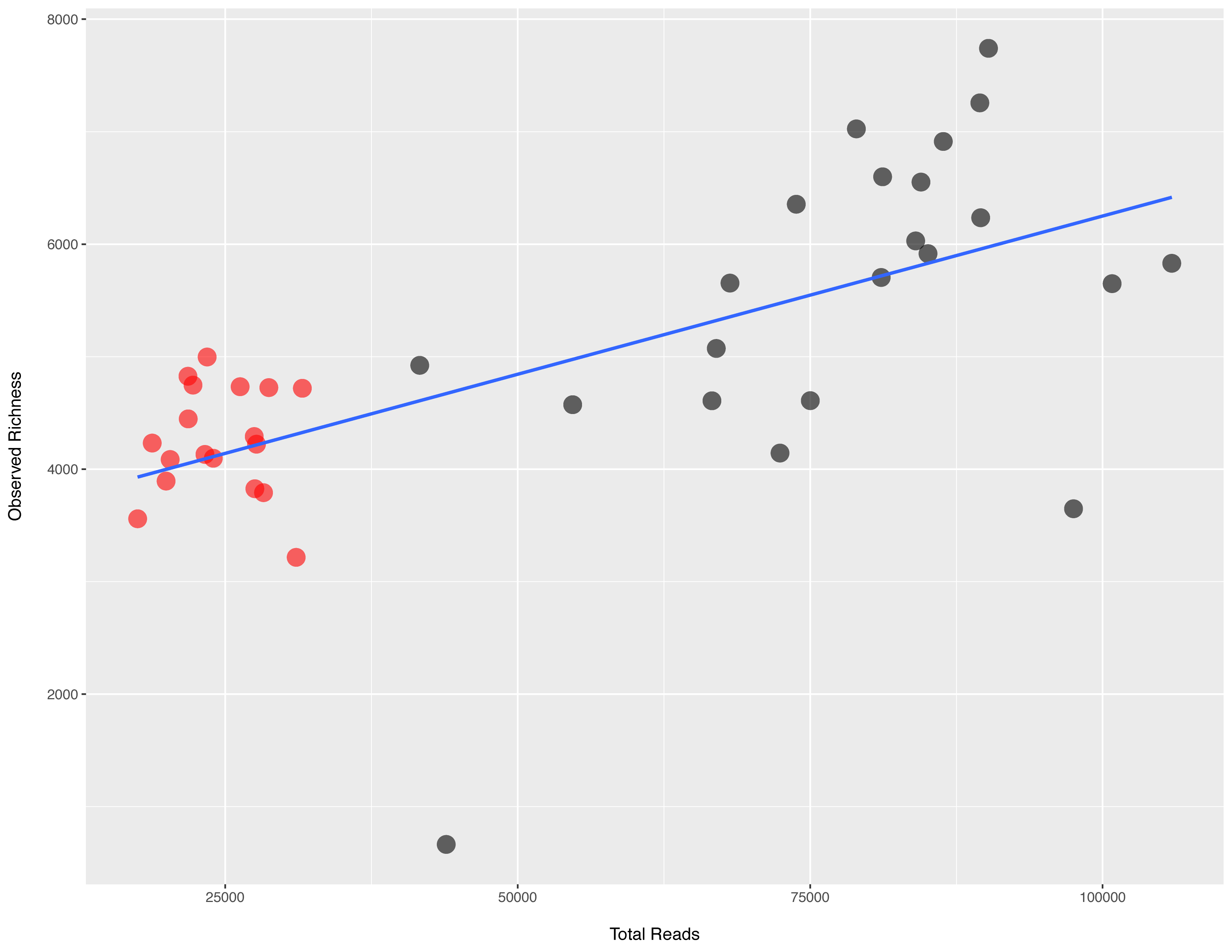

Supplement: S1 Fig — Observed richness (total number of OTUs) based on the total number of reads recovered per sample where color indicates the sequencing batch. There was a statistically significant Pearson’s correlation between the number of total reads and the observed richness; p < 0.001 and R2 = 0.63. (TIF) [file pone.0258079.s001.tif]

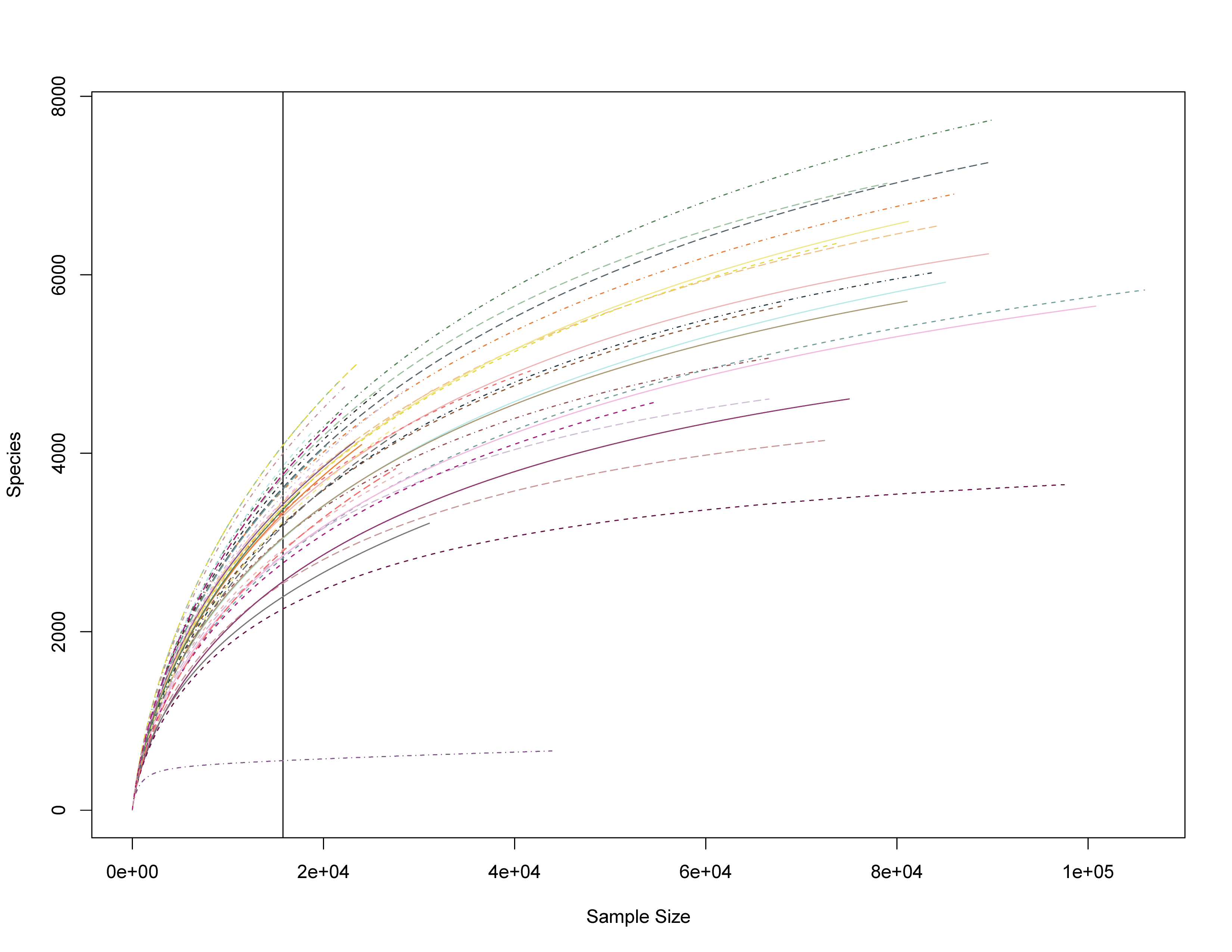

Supplement: S2 Fig — Rarefaction curves for all forty samples in the dataset. Where each curve indicates a different sample and the vertical line is the sampling depth of 15,771 reads. (TIF) [file pone.0258079.s002.tif]

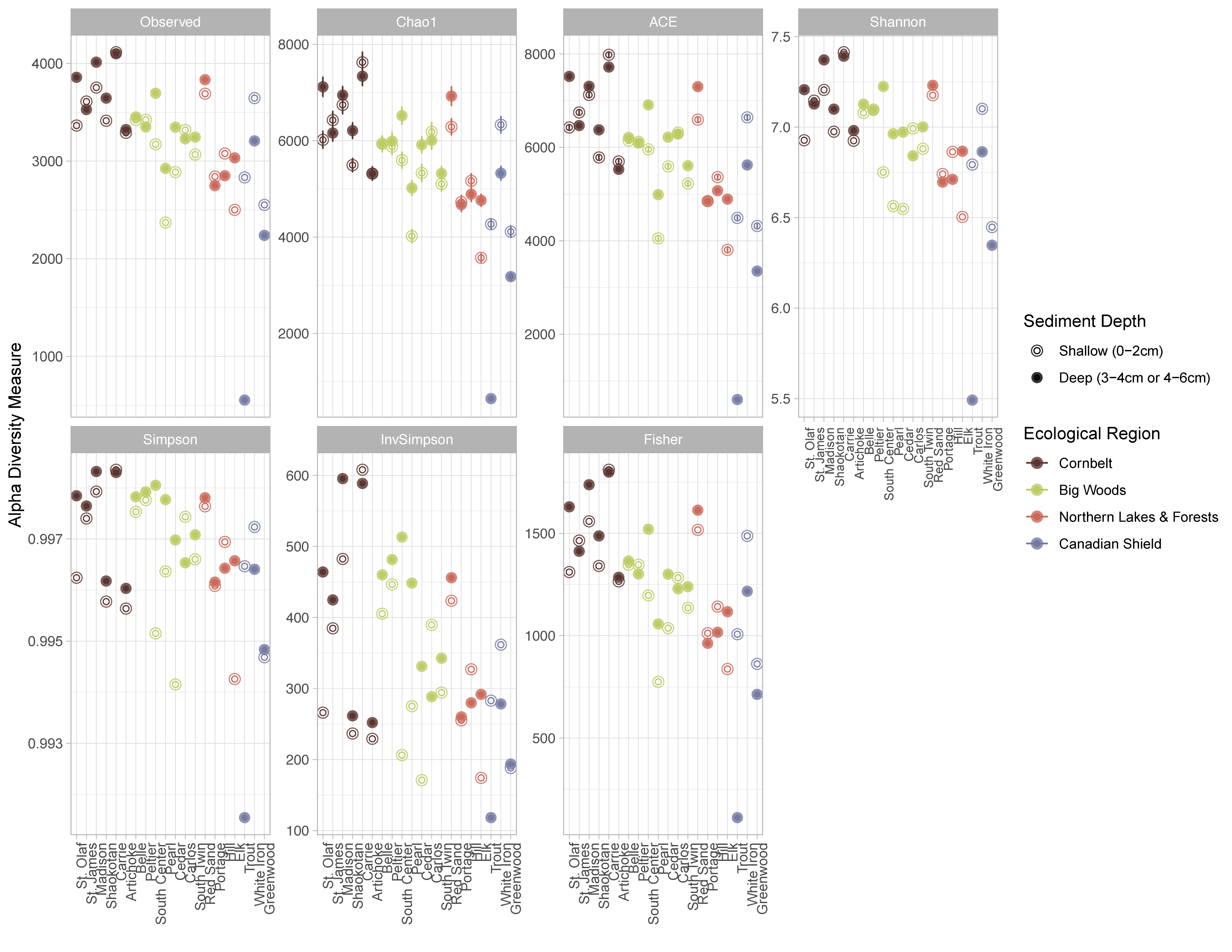

Supplement: S3 Fig — Alpha diversity measures across samples, where shape indicates depth of sample and color indicates ecological regions. All measures were calculated using Phyloseq and exhibit similar patterns in diversity; decreasing diversity across a northeasterly transect. (TIF) [file pone.0258079.s003.tif]

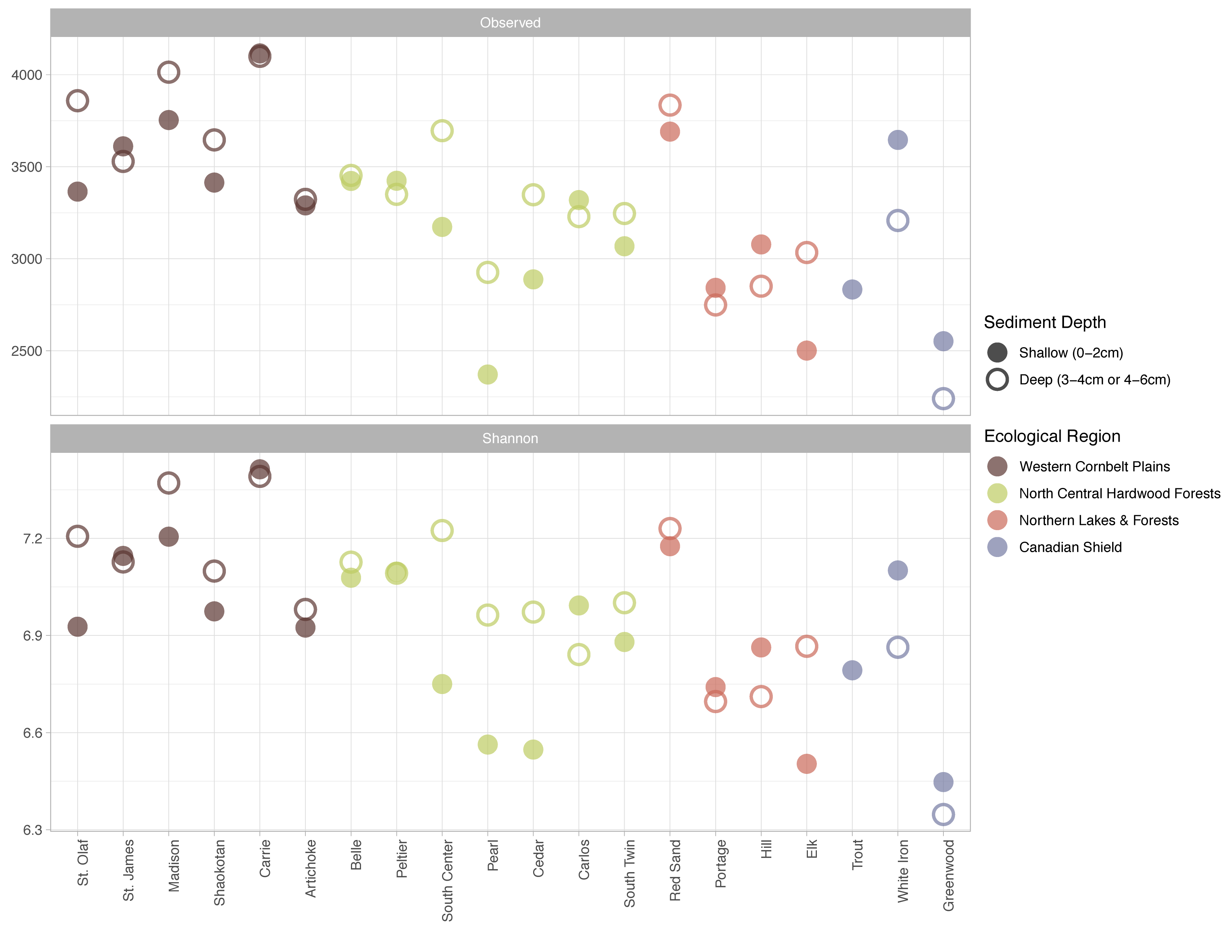

Supplement: S4 Fig — Observed diversity, a measure of richness, and Shannon diversity, measure of evenness, for all samples where shape indicates the sediment depth, color indicates the ecological region, and sites are ordered based on ecological region then latitude. One sample (Trout, Deep—CS), with lower diversity, was removed for visualization. (TIF) [file pone.0258079.s004.tif]

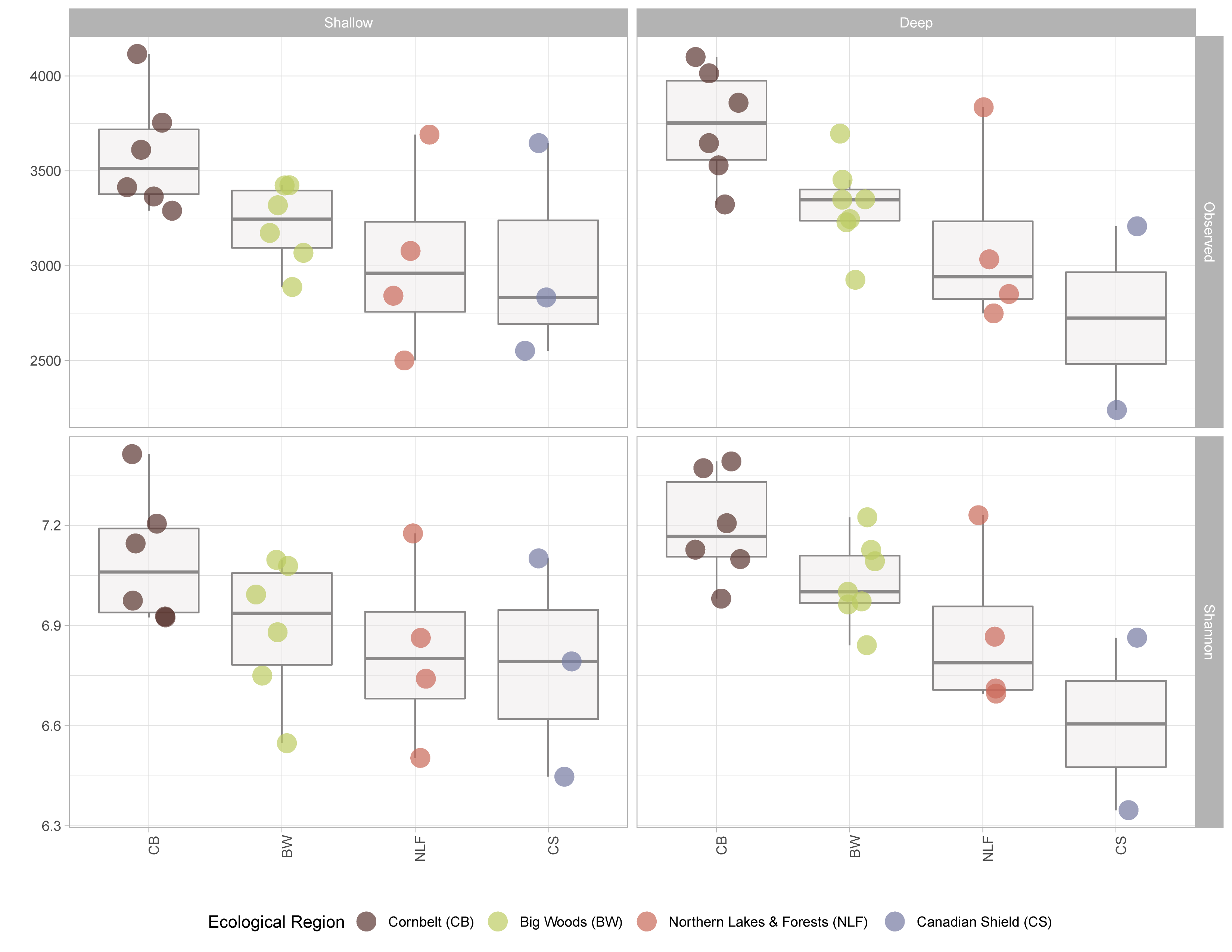

Supplement: S5 Fig — Boxplots show mean alpha level diversity of the Observed Operational Taxonomic Units (OTUs) and Shannon indices for the four ecological regions within the study area: Western Cornbelt Plains (CB), North Central Hardwood Forests (NCHF), Northern Lakes and Forests (NLF), and Canadian Shield (CS). Samples are faceted by their sediment depth where Shallow is 0-2cm deep and Deep is 3–4 or 4-6cm deep. One deep, CS sample was removed from alpha diversity metric both plots for due to uncharacteristically low diversity. (TIF) [file pone.0258079.s005.tif]

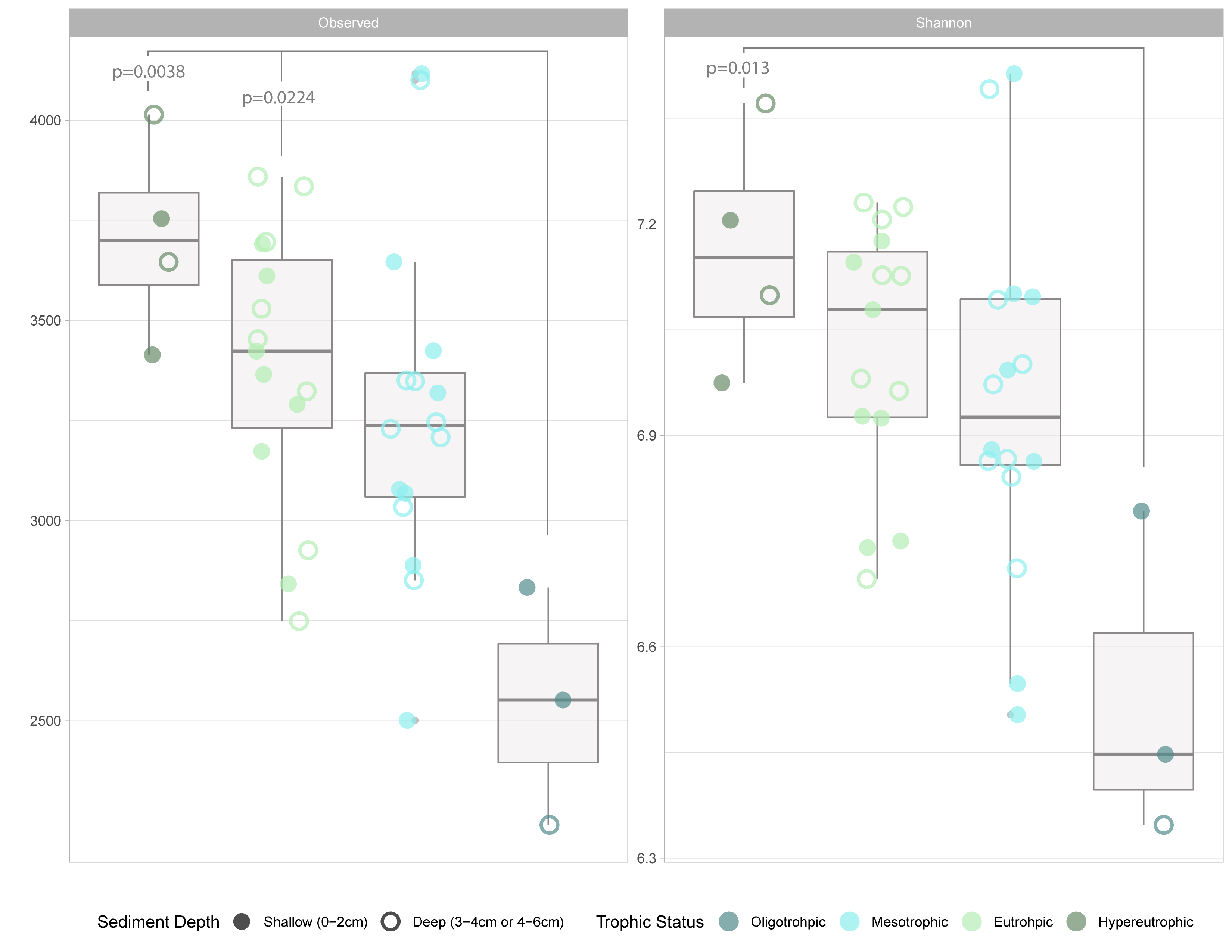

Supplement: S6 Fig — Box plots show mean alpha level diversity of the observed Operational Taxonomic Units (OTUs) and Shannon indices of the four trophic status classifications within the study area: Oligotrophic, Mesotrophic, Eutrophic, and Hypereutrophic. Each point represents a given sample where shape indicates depth of sample and color indicates the trophic status. One sample with extremely low richness and diversity was removed from both plots for visualization. Significance between regions was calculated nonparametrically using a Kruskal Wallis H test followed by a Dunn post hoc test with a Bonferroni correction. Reported p-values indicate significant differences in Observed and Shannon diversity, respectively, across trophic status, specifically the diversity of Oligotrophic lake sediments when compared to Eutrophic (p = 0.0224) and Hypereutrophic (p = 0.0038 & p = 0.013) sediments. (TIF) [file pone.0258079.s006.tif]

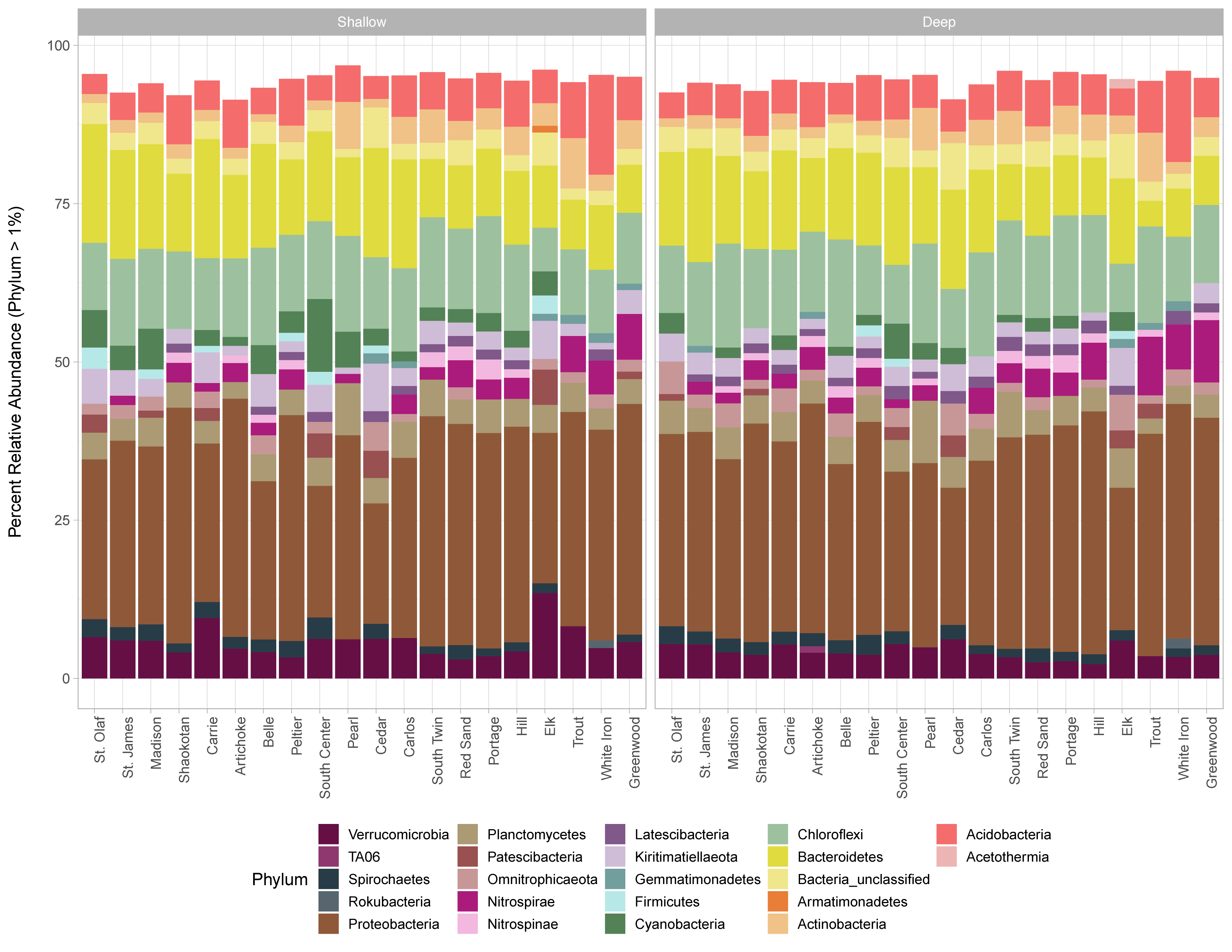

Supplement: S7 Fig — Bar plots of phyla that comprise >1% of the total relative abundance of a given sample. Samples are sorted along the X axis by ecological region. (TIF) [file pone.0258079.s007.tif]

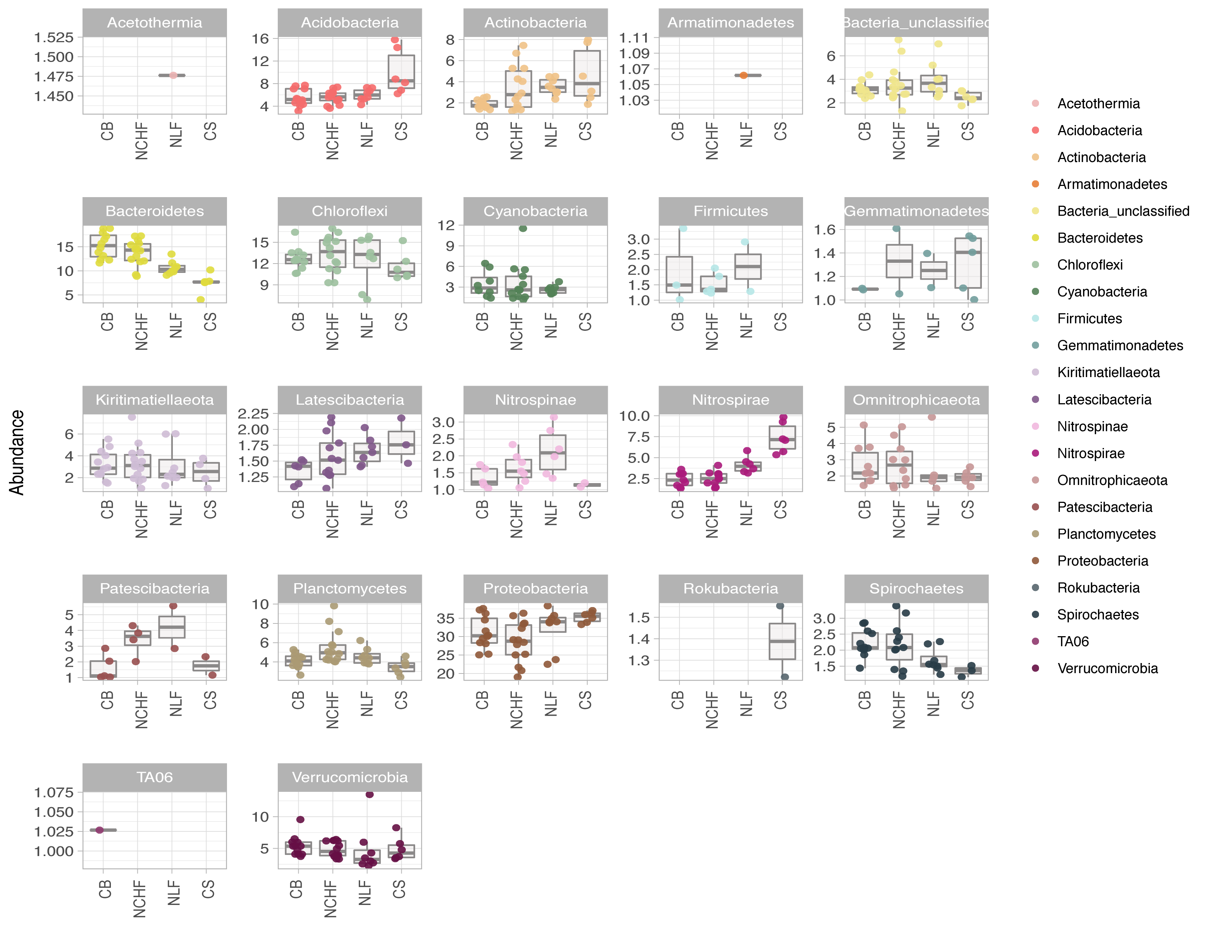

Supplement: S8 Fig — Box plots show mean relative abundance for the phyla across ecological region. Each point is a sample. Abbreviations: Western Cornbelt Plains (CB), North Central Hardwood Forests (NCHF), Northern Lakes and Forests (NLF), and Canadian Shield (CS). (TIF) [file pone.0258079.s008.tif]

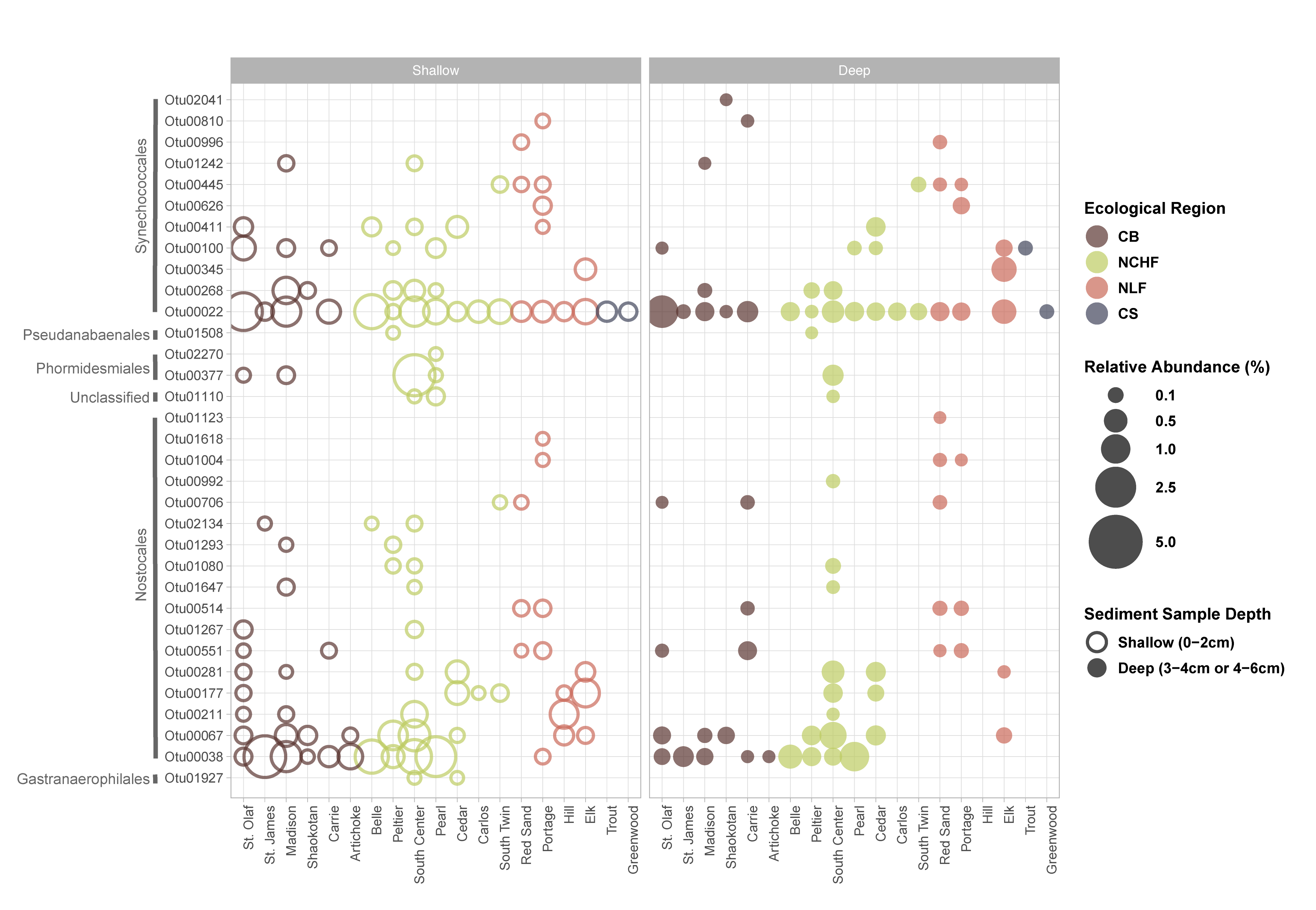

Supplement: S9 Fig — Abundance comparison of sediment cyanobacterial communities where shape indicates depth of samples, color ecological region, and size the relative abundance in percent. Bars along the left group the OTUs by order. OTUs were selected if they comprised >0.01% of the total relative abundance of the sample. (TIF) [file pone.0258079.s009.tif]
